# Modeling and Analysis of Energy Conservation Scheme Based on Duty Cycling in Wireless *Ad Hoc* Sensor Network

**DOI:** 10.3390/s100605569

**Published:** 2010-06-03

**Authors:** Yun Won Chung, Ho Young Hwang

**Affiliations:** 1 School of Electronic Engineering, Soongsil University, 511 Sangdo-dong, Dongjak-gu, Seoul, Korea; 2 Department of Electrical and Computer Engineering, University of Waterloo, Waterloo, ON N2L 3G1, Canada; E-Mail: hyhwang@bbcr.uwaterloo.ca

**Keywords:** sensor network, energy conservation, energy consumption, duty cycling, steady state probability

## Abstract

In sensor network, energy conservation is one of the most critical issues since sensor nodes should perform a sensing task for a long time (e.g., lasting a few years) but the battery of them cannot be replaced in most practical situations. For this purpose, numerous energy conservation schemes have been proposed and duty cycling scheme is considered the most suitable power conservation technique, where sensor nodes alternate between states having different levels of power consumption. In order to analyze the energy consumption of energy conservation scheme based on duty cycling, it is essential to obtain the probability of each state. In this paper, we analytically derive steady state probability of sensor node states, *i.e.*, *sleep*, *listen*, and *active* states, based on traffic characteristics and timer values, *i.e.*, *sleep timer*, *listen timer*, and *active timer*. The effect of traffic characteristics and timer values on the steady state probability and energy consumption is analyzed in detail. Our work can provide sensor network operators guideline for selecting appropriate timer values for efficient energy conservation. The analytical methodology developed in this paper can be extended to other energy conservation schemes based on duty cycling with different sensor node states, without much difficulty.

## 1. Introduction

Sensor network generally consists of multiple sensor nodes and a sink node, where sensor nodes monitor and measure physical information such as temperature, humidity, pressure, movement of any object, *etc.* [1, 2]. Sensed information is transferred from sensor nodes to a sink node via multi-hop routing and it is finally delivered to the central management system.

In sensor network, sensor nodes should perform a sensing task for a long time (e.g., lasting a few years) and the battery of them cannot be replaced in most practical situations. Therefore, energy conservation or power control is considered as one of the most critical issues in sensor network in order to guarantee a certain level of performance [3]. It is also widely accepted that energy consumption due to communication is much higher than that due to either sensing information or processing sensed information [4, 5]. Regarding energy consumption due to communication, just listening communication interface also consumes comparable power to receiving data and it is reported that the ratio of energy consumption among listening, receiving, and transmitting data is about 1, 1, and 1.5, respectively [5, 6]. Therefore, it is important to put communication interface of sensor nodes in low power *sleep* state when communication between neighbor sensor nodes is not required, in order to save energy [7].

In order to extend the lifetime of sensor nodes, numerous works have been carried out and most algorithms proposed in these works mainly use duty cycling, data-driven approaches, and mobility [4]. Duty cycling achieves energy conservation by putting unused communication interface of sensor nodes in low power *sleep* state as long as possible. Data-driven approaches reduce the number of sampled data by eliminating redundant data delivery to the sink node using correlation property between sampled data [8]. The energy consumption of sensor nodes can be reduced further if mobile nodes moving around sensor network can be used for data collection, and thus, sensor nodes just route their data to the mobile nodes via direct or limited multi-hop routing [9].

Out of these schemes, duty cycling scheme is considered the most suitable power conservation technique [4] and we only focus on energy conservation schemes based on duty cycling in this paper. In [4], the authors classify duty cycling as topology control and power management. In topology control, redundancy of sensor nodes is used and only a subset of sensor nodes are activated, while maintaining network connectivity, and the other sensor nodes are deactivated for power saving [10–12]. Thus, it is important to decide which sensor nodes should be activated or deactivated in topology control.

Power management deals with activated sensor nodes and transitions between states having different levels of power consumption, such as *sleep*, *listen*, and *active* states, are controlled for efficient power conservation [13–15]. In power management, sensor nodes initially stay in *sleep* state when communication is not required, by putting communication interface in the low power *sleep* state. In order to check any incoming data, a sensor node periodically wakes up at the expiration of *sleep timer* by moving into *listen* state and listens to the radio interface. In *listen* state, if there is no incoming data until the expiration of *listen timer*, it moves back to *sleep* state. Otherwise, its state changes to *active* state and communication with another sensor node is performed. In *active* state, if there is no further data to be transmitted or received until the expiration of *active timer*, after completing transmitting or receiving any data, it moves to *sleep* state again.

In duty cycling, since different states of sensor nodes have different levels of power consumption, it is essential to obtain the steady state probability of sensor node states in order to analyze the energy consumption [3]. Also, since state transitions are controlled by timer values, the effect of timer values and traffic characteristics on the steady state probability should be analyzed in detail, so that sensor network operators can select appropriate timer values of sensor nodes for efficient energy conservation. To the best of our knowledge, however, energy consumption in most previous works is analyzed via simulation, without deriving the steady state probability analytically. Furthermore, the effect of timer values on energy consumption has not been investigated analytically in detail. In this sense, our work is the first attempt to derive the steady state probability of sensor node states based on traffic characteristics and timer values and investigate the effect of traffic characteristics and timer values on energy consumption analytically. We note that since the analytical methodology developed in this paper is based on a general energy conservation scheme based on duty cycling, it can be extended to other energy conservation schemes based on duty cycling with different sensor node states, without much difficulty.

The remainder of this paper is organized as follows: Section 2 surveys related works on duty cycling. Section 3 develops analytical model for deriving steady state probability of sensor node states and obtains energy consumption. Numerical examples are presented in Section 4. Finally, Section 5 summarizes this work and presents further works.

## 2. Related Works

In this section, related works on energy conservation schemes based on duty cycling are surveyed. As mentioned in Introduction, duty cycling can be classified as topology control and power management. In addition, topology control is further divided into location driven protocol and connectivity driven protocol based on the criteria used to decide activation and deactivation of sensor nodes [4]. In location driven protocol, the location of sensor nodes is used as the criteria and geographical adaptive fidelity (GAF) [11] is a representative example of this protocol. In connectivity driven protocol, on the other hand, network connectivity is used as the criteria and Span [12] is one example of this protocol.

In GAF [11], sensor network is divided into grids, where any two sensor nodes located within adjacent grids can communicate with each other. In each grid, only one sensor node is active and other sensor nodes in the grid are in *sleep* state. There are three states in GAF, *i.e.*, *sleep*, *discovery*, and *active* states. In *sleep* state, radio interface of a sensor node is put into *sleep* state and the sensor node moves into *discovery* state at the expiration of *sleep timer*. In *discovery* state, the sensor node exchanges discovery messages with other sensor nodes within the same grid. If the sensor node receives a discovery message from a sensor node with higher rank, *i.e.*, higher residual energy, within *discovery timer*, it moves back to *sleep* state. Otherwise, it moves to *active* state. In *active* state, if the sensor node receives a discovery message from a sensor node with higher rank within *active timer*, it enters into *sleep* state. Otherwise, it moves back to *discovery* state at the expiration of *active timer*.

Span [12] is a distributed backbone selection protocol and adaptively elects coordinators in sensor network. The coordinators perform multi-hop routing for other sleeping sensor nodes by staying awake. Sensor nodes in *sleep* state periodically wake up and check whether to sleep or stay awake as a coordinator using coordinator eligibility rule which is based on the battery level and the number of neighbor sensor nodes. In coordinator eligibility rule, if two neighbor sensor nodes of a non-coordinator sensor node cannot reach each other, either directly or via one or two coordinators, the non-coordinator sensor node becomes a coordinator sensor node. In order to avoid the situation that multiple sensor nodes become a coordinator at the same time, a random back off delay is used before taking a coordinator role. Each coordinator periodically checks if it can withdraw a coordinator role by checking if every pair of sensor nodes of its neighbor sensor nodes can communicate, either directly or via one or two coordinators.

In power management, activated sensor nodes alternate between *sleep*, *listen*, and *active* states, based on traffic characteristics, without depending on topology or connectivity information. The power management scheme is further divided into sleep/wakeup protocol which is independent on medium access control (MAC) protocol and MAC protocol with low duty cycle, where sleep/wakeup is tightly coupled with MAC protocol [4]. In this paper, we only focus on sleep/wakeup protocol because it can be used with any MAC protocol. Basic energy conservation algorithm (BECA)[13] and sparse topology and energy management (STEM) [14] belong to this protocol.

**Figure 1.**
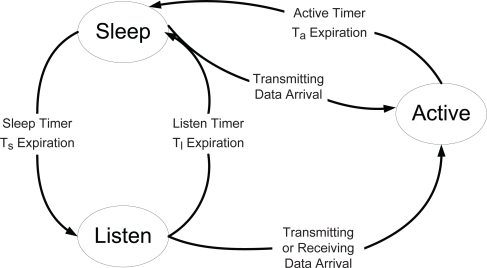
State transition model of BECA.

In BECA [13], sensor nodes stay in *sleep* state initially when communication is not required, by putting the communication interface in the low power *sleep* state. Each sensor node periodically wakes up for every *sleep timer*, *T_s_*, by moving into *listen* state and listens any incoming data to the sensor node during *listen timer*, *T_l_*. In *listen* state, if there is no incoming data until the expiration of the *listen timer*, it moves back to *sleep* state again. Otherwise, its state changes to *active* state and it communicates with another sensor node. In *active* state, if there is no further data to be transmitted or received until the expiration of *active timer*, *T_a_*, after completing transmitting or receiving any data, it moves to *sleep* state again. Figure 1 shows state transition model of BECA.

In STEM [14], all sensor nodes wake up simultaneously for every *T_wakeup_* and remain in *active* state for active time. Then, they move to *sleep* state at the same time until the next wakeup time. In STEM, low duty cycle, *i.e.*, high energy efficiency, is possible by adjusting active time much smaller than wakeup time. The STEM has the problem of high probability of collisions due to simultaneous wakeup and thus, staggered wakeup pattern (SWP) [15] has been proposed to solve this problem by waking up sensor nodes at different times based on the different levels of routing tree.

## 3. Modeling and Analysis of Sensor Node State Transition Model

In this paper, we develop an analytical methodology for deriving steady state probability of sensor node states of BECA as an example. We note that the analytical methodology developed for BECA can be extended to other energy conservation schemes based on duty cycling with different sensor node states, without much difficulty.

### 3.1. Modeling of Sensor Node State Transition

**Figure 2.**
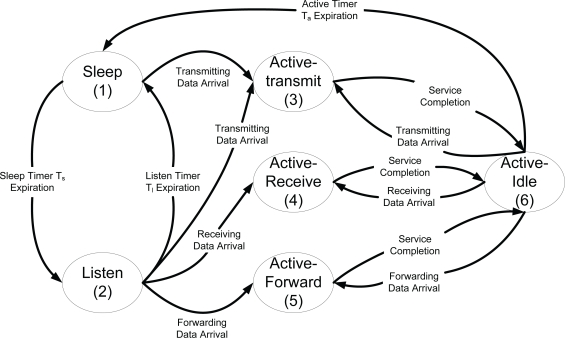
A modified state transition model of BECA.

Figure 2 shows a modified state transition model of BECA, where *active* state in original state transition model of BECA is divided into four sub-states; *active-transmit*, *active-receive*, *active-forward*, and *active-idle* states, for ease of mathematical derivation. In *active-transmit* state, a sensor node transmits its locally generated sensing data to a sink node. In *active-forward* state, on the other hand, a sensor node relays sensing data from other sensor nodes to neighbor sensor nodes. In *active-receive* state, a sensor node receives sensing data from other sensor nodes. In *active-idle* state, the sensor node does not receive or transmit any sensing data, and if there is no further data to be transmitted or received until the expiration of *active timer*, it moves to *sleep* state. We note that we differentiate *active-forward* state from *active-transmit* state because traffic characteristic for a sensor node’s locally generated sensing data traffic is different from aggregated sensing data generated by other sensor nodes, and thus, we need to consider them separately for an accurate modeling [16].

### 3.2. Derivation of Steady State Probability

For mathematical derivation, we need to assume traffic model of sensor networks, which depends on the data reporting process of considered sensor network applications, *i.e.*, event-driven, time-driven, and query-driven [17, 18]. In event-driven case, data are generated when an event occurs. In time-driven case, data are generated periodically. In query-driven case, data are generated by the request of a sink node when needed. In this paper, we mainly focus on event-driven case and it is proved that the data generation at an individual sensor node follows a Poisson process, based on the assumptions that the event is uniformly distributed in sensor networks and every event is independent of other events [17]. Regarding the service time of data packet, we use an exponential distribution model for more general consideration of service time, although simple constant bit rate (CBR) model is commonly used. The use of Poisson and exponential distributions allows us to develop a tractable analytical model for deriving steady state probability of sensor node states. The developed model based on Poisson and exponential distributions can be easily extended to other cases with different traffic model.

Based on the above discussions, we have made the following assumptions regarding the density functions of random variables:

- Transmitting, receiving, and forwarding data packets at a sensor node occur according to a Poisson process with parameters *λ_t_*, *λ_r_*, and *λ_f_*, respectively;
- The time duration that a sensor node remains in *active-transmit*, *active-receive*, and *active-forward* states follows an exponential distribution with a mean value of 1 / *μ_t_*, 1 / *μ_r_*, and 1 / *μ_f_* ;
- The values of *sleep timer*, *listen timer*, and *active timer* are assumed as constant and they are denoted by *T_s_*, *T_l_*, and *T_a_*, respectively.

We denote *sleep*, *listen*, *active-transmit*, *active-receive*, *active-forward*, and *active-idle* states as states 1, 2, 3, 4, 5, and 6, respectively, for notational convenience. Since the residence times of a sensor node in *sleep*, *listen*, and *active-idle* states do not follow an exponential distribution, the sensor node state transition behavior is analyzed using a semi-Markov process approach [19].

The steady state probability of each sensor node state can be obtained as [19]:

(1) $$P_{k}=\frac{\pi_{k}\bar{t_{k}}}{\sum_{i=1}^{6}\pi_{i}\bar{t_{i}}},\;\;\;\;\;\;\;k=1,2,3,4,5,\;\text{and}\;6$$

where *π_k_* denotes the stationary probability of state *k* and $\bar{t_{k}}$ is the mean residence time of the sensor node in state *k*. The stationary probability is obtained by solving the following balancing equations [19]:

(2) $$\pi_{j}=\sum_{k=1}^{6}\pi_{k}P_{\mathrm{kj}},\;j=1,2,3,4,5,\;\text{and}\;6$$

(3) $$1=\sum_{k=1}^{6}\pi_{k}\;$$

where *P_kj_* represents the state transition probability from state *k* to state *j*. The state transition probability matrix *P* = [*p_kj_*] of the state transition model is given by:

$$P=\left(\begin{array}{cccccc} 0 & P_{12} & P_{13} & 0 & 0 & 0\\ P_{21} & 0 & P_{23} & P_{24} & P_{25} & 0\\ 0 & 0 & 0 & 0 & 0 & P_{36}\\ 0 & 0 & 0 & 0 & 0 & P_{46}\\ 0 & 0 & 0 & 0 & 0 & P_{56}\\ P_{61} & 0 & P_{63} & P_{64} & P_{65} & 0 \end{array}\right)$$

Then, stationary probabilities can be solved as:

(4) $$\pi_{1}=\frac{1}{D}$$

(5) $$\pi_{2}=P_{12}\pi_{1}=\frac{P_{12}}{D}$$

(6) $$\pi_{3}=\frac{P_{61}P_{13}+P_{61}P_{12}P_{23}+P_{63}(1-P_{12}P_{21})}{P_{61}}\pi_{1}=\frac{P_{61}P_{13}+P_{61}P_{12}P_{23}+P_{63}(1-P_{12}P_{21})}{P_{61}D}$$

(7) $$\pi_{4}=\frac{P_{61}P_{12}P_{24}+P_{64}(1-P_{12}P_{21})}{P_{61}}\pi_{1}=\frac{P_{61}P_{12}P_{24}+P_{64}(1-P_{12}P_{21})}{P_{61}D}$$

(8) $$\pi_{5}=\frac{P_{61}P_{12}P_{25}+P_{65}(1-P_{12}P_{21})}{P_{61}}\pi_{1}=\frac{P_{61}P_{12}P_{25}+P_{65}(1-P_{12}P_{21})}{P_{61}D}$$

(9) $$\pi_{6}=\frac{1-P_{12}P_{21}}{P_{61}}\pi_{1}=\frac{1-P_{12}P_{21}}{P_{61}D}$$

where *D* is obtained by:

(10) $$D=1+P_{12}(1+P_{23}+P_{24}+P_{25})+P_{13}+\frac{1+P_{63}+P_{64}+P_{65}-P_{12}P_{21}(1+P_{63}+P_{64}+P_{65})}{P_{61}}$$

State transition probability *P_kj_* can be derived based on the distribution of time from states *k* to *j*, *T_kj_*. Exit from the *sleep* state is caused by any of the following events:

- *Sleep* timer expiration (*T*_12_);
- A transmitting data packet arrival (*T*_13_).

Then, the state transition probabilities *P*_12_ and *P*_13_ are obtained as:

(11) $$\begin{array}{lll} P_{12} & = & \int_{0}^{\infty }f_{T_{12}}(t)\mathrm{Pr}(T_{13}>t)\mathrm{dt}=\int_{0}^{\infty }\delta (t-T_{s})\int_{0}^{\infty }\lambda_{t}\;e^{-\lambda_{t}\;u}\mathrm{dudt}\\ & = & \int_{0}^{\infty }\delta (t-T_{s})e^{-\lambda_{t}\;t}\mathrm{dt}=e^{-\lambda_{t}\;T_{s}} \end{array}$$

(12) $$P_{13}=1-P_{12}=1-e^{-\lambda_{t}T_{s}}$$

Exit from the *listen* state is caused by any of the following events:

- *Listen* timer expiration (*T*_21_);
- A transmitting data packet arrival (*T*_23_);
- A receiving data packet arrival (*T*_24_);
- A forwarding data packet arrival (*T*_25_).

Then, the state transition probability *P*_21_ is obtained as:

(13) $$\begin{array}{lll} P_{21} & = & \int_{0}^{\infty }f_{T_{21}}(t)\mathrm{Pr}(T_{23}>t)\mathrm{Pr}(T_{24}>t)\mathrm{Pr}(T_{25}>t)\mathrm{dt}\\ & = & \int_{0}^{\infty }\delta (t-T_{l})\int_{t}^{\infty }\lambda_{t}e^{-\lambda_{t}u}\mathrm{du}\int_{t}^{\infty }\lambda_{r}e^{-\lambda_{r}u}\mathrm{du}\int_{t}^{\infty }\lambda_{f}e^{-\lambda_{f}\;u}\mathrm{dudt}\\ & = & \int_{0}^{\infty }\delta (t-T_{l})e^{-\lambda_{t}t}e^{-\lambda_{r}t}e^{-\lambda_{f}\;t}\mathrm{dt}=e^{(-\lambda_{t}+\lambda_{r}+\lambda_{f})T_{t}} \end{array}$$

Similarly, the state transition probabilities *P*_23_, *P*_24_, and *P*_25_ are obtained as:

(14) $$\begin{array}{lll} P_{23} & = & \int_{0}^{\infty }f_{T_{23}}(t)\mathrm{Pr}(T_{21}>t)\mathrm{Pr}(T_{24}>t)\mathrm{Pr}(T_{25}>t)\mathrm{dt}\\ & = & \frac{\lambda_{t}}{\lambda_{t}+\lambda_{r}+\lambda_{f}}(1-e^{(-\lambda_{t}+\lambda_{r}+\lambda_{f})T_{l}}) \end{array}$$

(15) $$\begin{array}{lll} P_{24} & = & \int_{0}^{\infty }f_{T_{24}}(t)\mathrm{Pr}(T_{21}>t)\mathrm{Pr}(T_{23}>t)\mathrm{Pr}(T_{25}>t)\mathrm{dt}\\ & = & \frac{\lambda_{r}}{\lambda_{t}+\lambda_{r}+\lambda_{f}}(1-e^{(-\lambda_{t}+\lambda_{r}+\lambda_{f})T_{l}}) \end{array}$$

(16) $$\begin{array}{lll} P_{25} & = & \int_{0}^{\infty }f_{T_{25}}(t)\mathrm{Pr}(T_{21}>t)\mathrm{Pr}(T_{23}>t)\mathrm{Pr}(T_{24}>t)\mathrm{dt}\\ & = & \frac{\lambda_{f}}{\lambda_{t}+\lambda_{r}+\lambda_{f}}(1-e^{(-\lambda_{t}+\lambda_{r}+\lambda_{f})T_{l}}) \end{array}$$

In *active-transmit*, *active-receive*, and *active-forward* states, exit from these states are caused by the service completion of transmitting, receiving, and forwarding data packets, respectively, and the state transition probabilities *P*_36_, *P*_46_, and *P*_56_ are simply obtained as:

(17) $$P_{36}=P_{46}=P_{56}=1$$

Exit from the *active-idle* state is caused by any of the following events:

- *Active* timer expiration (*T*_61_);
- A transmitting data packet arrival (*T*_63_);
- A receiving data packet arrival (*T*_64_);
- A forwarding data packet arrival (*T*_65_).

Then, the state transition probability *P*_61_ is obtained as:

(18) $$\begin{array}{lll} P_{61} & = & \int_{0}^{\infty }f_{T_{61}}(t)\mathrm{Pr}(T_{63}>t)\mathrm{Pr}(T_{64}>t)\mathrm{Pr}(T_{65}>t)\mathrm{dt}\\ & = & \int_{0}^{\infty }\delta (t-T_{a})\int_{t}^{\infty }\lambda_{t}e^{-\lambda_{t}u}\mathrm{du}\int_{t}^{\infty }\lambda_{r}e^{-\lambda_{r}u}\mathrm{du}\int_{t}^{\infty }\lambda_{f}e^{-\lambda_{f}\;u}\mathrm{dudt}\\ & = & \int_{0}^{\infty }\delta (t-T_{a})e^{-\lambda_{t}t}e^{-\lambda_{r}t}e^{-\lambda_{f}\;t}\mathrm{dt}=e^{(-\lambda_{t}+\lambda_{r}+\lambda_{f})T_{a}} \end{array}$$

Similarly, the state transition probabilities *P*_63_, *P*_64_, and *P*_65_ are obtained as:

(19) $$\begin{array}{lll} P_{63} & = & \int_{0}^{\infty }f_{T_{63}}(t)\mathrm{Pr}(T_{61}>t)\mathrm{Pr}(T_{64}>t)\mathrm{Pr}(T_{65}>t)\mathrm{dt}\\ & = & \frac{\lambda_{t}}{\lambda_{t}+\lambda_{r}+\lambda_{f}}(1-e^{(-\lambda_{t}+\lambda_{r}+\lambda_{f})T_{a}}) \end{array}$$

(20) $$\begin{array}{lll} P_{64} & = & \int_{0}^{\infty }f_{T_{64}}(t)\mathrm{Pr}(T_{61}>t)\mathrm{Pr}(T_{63}>t)\mathrm{Pr}(T_{65}>t)\mathrm{dt}\\ & = & \frac{\lambda_{r}}{\lambda_{t}+\lambda_{r}+\lambda_{f}}(1-e^{(-\lambda_{t}+\lambda_{r}+\lambda_{f})T_{a}}) \end{array}$$

(21) $$\begin{array}{lll} P_{65} & = & \int_{0}^{\infty }f_{T_{65}}(t)\mathrm{Pr}(T_{61}>t)\mathrm{Pr}(T_{63}>t)\mathrm{Pr}(T_{64}>t)\mathrm{dt}\\ & = & \frac{\lambda_{f}}{\lambda_{t}+\lambda_{r}+\lambda_{f}}(1-e^{(-\lambda_{t}+\lambda_{r}+\lambda_{f})T_{a}}) \end{array}$$

Now, we have derived all the state transition probabilities, and the mean residence time of the sensor node in each state is calculated. The mean residence time in the *sleep* state is derived as:

(22) $$\begin{array}{lll} \bar{t_{1}} & = & E[t_{1}]=E[\text{min}\{T_{12},\;T_{13}\}]=\int_{0}^{\infty }\text{Pr}(\text{min}\{T_{12},\;T_{13}\}>t)\mathrm{dt}\\ & = & \int_{0}^{\infty }\text{Pr}(T_{12}>t)\text{Pr}(T_{13}>t)\mathrm{dt}=\int_{0}^{\infty }U[T_{s}-t]e^{-\lambda_{t}t}\mathrm{dt}=\int_{0}^{T_{s}}e^{-\lambda_{t}t}\mathrm{dt}=\frac{1-e^{-\lambda_{t}T_{s}}}{\lambda_{t}} \end{array}$$

The mean residence time in the *listen* state is similarly derived as:

(23) $$\begin{array}{lll} \bar{t_{2}} & = & E[t_{2}]=E[\text{min}\{T_{21},\;T_{23},T_{24},T_{25}\}]=\int_{0}^{\infty }\text{Pr}(\text{min}\{T_{21},\;T_{23},T_{24},T_{25}\}>t)\mathrm{dt}\\ & = & \int_{0}^{\infty }\text{Pr}(T_{21}>t)\text{Pr}(T_{23}>t)\;\text{Pr}(T_{24}>t)\;\text{Pr}(T_{25}>t)\mathrm{dt}\\ & = & \int_{0}^{\infty }e^{-\lambda_{t}}{}^{t}\text{Pr}(T_{21}>t)\text{Pr}(T_{24}>t)\text{Pr}(T_{25}>t)\mathrm{dt}\\ & = & \frac{1}{\lambda_{t}}P_{23}=\frac{1}{\lambda_{t}+\lambda_{r}+\lambda_{f}}(1-e^{(-\lambda_{t}+\lambda_{r}+\lambda_{f})T_{l}}) \end{array}$$

The mean residence time in the *active-transmit* state is derived as:

(24) $$\bar{t_{3}}=E[t_{3}]=E[\text{min}\{T_{36}\}]=\int_{0}^{\infty }\text{Pr}(\text{min}\{T_{36}\}>t)\mathrm{dt}=\int_{0}^{\infty }e^{-\mu_{t}t}\mathrm{dt}=\frac{1}{\mu_{t}}$$

Likewise, the mean residence time in the *active-receive* and *active-forward* is derived as:

(25) $$\bar{t_{4}}=E[T_{4}]=E[\text{min}\{T_{46}\}]=\int_{0}^{\infty }\text{Pr}(\text{min}\{T_{46}\}>t)\mathrm{dt}=\int_{0}^{\infty }e^{-\mu_{t}t}\mathrm{dt}=\frac{1}{\mu_{r}}$$

(26) $$\bar{t_{5}}=E[t_{4}]=E[\text{min}\{T_{46}\}]=\int_{0}^{\infty }\text{Pr}(\text{min}\{T_{46}\}>t)\mathrm{dt}=\int_{0}^{\infty }e^{-\mu_{t}t}\mathrm{dt}=\frac{1}{\mu_{f}}$$

The mean residence time in the *active-idle* state is derived as:

(27) $$\begin{array}{lll} \bar{t_{6}} & = & E[t_{6}]=E[\text{min}\{T_{61},\;T_{63},T_{64},T_{65}\}]=\int_{0}^{\infty }\text{Pr}(\text{min}\{T_{61},\;T_{63},T_{64},T_{65}\}>t)\mathrm{dt}\\ & = & \int_{0}^{\infty }\text{Pr}(T_{61}>t)\text{Pr}(T_{63}>t)\;\text{Pr}(T_{64}>t)\;\text{Pr}(T_{65}>t)\mathrm{dt}\\ & = & \int_{0}^{\infty }e^{-\lambda_{t}t}\text{Pr}(T_{61}>t)\text{Pr}(T_{64}>t)\text{Pr}(T_{65}>t)\mathrm{dt}\\ & = & \frac{1}{\lambda_{t}}P_{63}=\frac{1}{\lambda_{t}+\lambda_{r}+\lambda_{f}}(1-e^{(-\lambda_{t}+\lambda_{r}+\lambda_{f})T_{a}}) \end{array}$$

Based on the values of *π_k_* and $\bar{t_{k}}$ obtained from Equations (4)–(9) and Equations (22)–(27), respectively, we can finally derive the steady state probability of each sensor node state using Equation (1) [19]. Now, we can obtain the energy consumption of a sensor node per unit time by using the steady state probability as:

(28) $$E=\sum_{k=1}^{6}\psi_{k}P_{k}$$

where *ψ_k_* is the power consumption in state *k*.R

## 4. Numerical Examples

### 4.1. Parameters Setting

In this section, numerical examples are presented in order to show the effect of various parameters on the steady state probability and energy consumption. In sensor network, since sensor nodes transmit locally generated sensing data to a sink node and the locally generated data traffic from sensor nodes contributes to forwarding data traffic of a sensor node, we relate local transmitting data traffic and forwarding data traffic of a sensor node by *λ_f_* = *w_f_λ_t_*, where *w_f_* is the weighting factor for forwarding data traffic to local transmitting data traffic and it is proportional to the number of sensor nodes in sensor network. Also, since forwarding traffic should be received first before forwarding [16], the relationship *λ_r_* = *λ_f_* is assumed. The relationship 1/*μ_t_* = 1/*μ_r_* = 1/*μ_f_* is also assumed since each sensor node normally generates sensing data with the same size. Based on these relationships, we define activity of a sensor node as follows:

(29) $$\rho =\frac{\lambda_{t}+\lambda_{r}+\lambda_{f}}{\mu_{t}}=\frac{(1+{2w}_{f})\lambda_{t}}{\mu_{t}}$$

The default parameters for timers and traffic characteristic are given by Table 1. Parts of the parameters in Table 1 vary in the following numerical examples in order to analyze the effect of the parameters on the performance. Power consumption in each state is assumed, as the ones depicted in Table 2, based on the energy model described in [6, 11, 12].

### 4.2. Effect of Sleep Timer, T_s_

**Figure 3.**
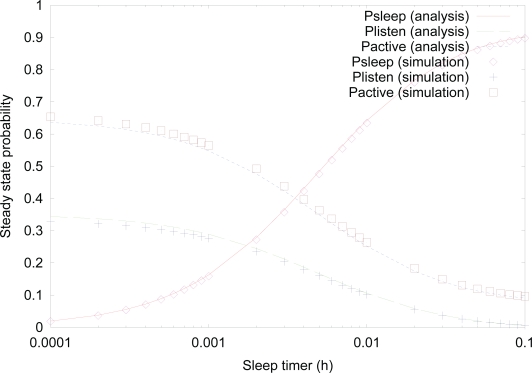
Effect of *sleep timer* on the steady state probability.

Figure 3 shows the effect of *sleep timer* on the steady state probability of *sleep*, *listen*, and *active* states. We note that instead of showing the probabilities of four sub-states; *active-transmit*, *active-receive*, *active-forward*, and *active-idle* states which have a very similar pattern, we just show the probability of *active* state collectively, in order to avoid confusion and strengthen the result. To validate the analytical methodology developed in this paper, we conducted a discrete-event simulation for steady state probabilities, using C++ language. The simulation results were obtained by running 10^4^ h of simulation time after 10^3^ hours of warm-up time. From the results, it is shown that the validity of the proposed analytical model is proved since analysis results are well matched with simulation results. As the value of *sleep timer* increases, the probability of *sleep* state decreases, since it is more likely that a sensor node stays in *sleep* state more for higher value of *sleep timer*. On the other hand, the probabilities of *listen* state and *active* state decrease, because the chance of staying in either *listen* state or *active* state decreases due to increased probability of *sleep* timer.

**Figure 4.**
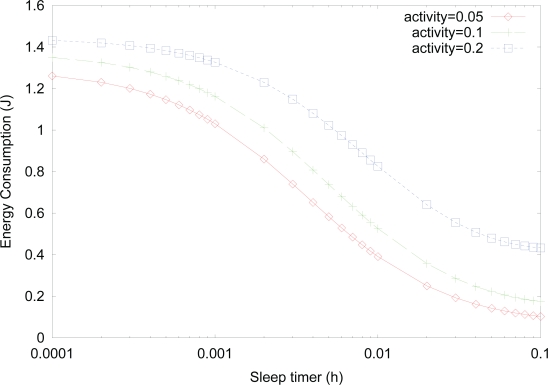
Effect of *sleep timer* on the energy consumption for varying activity.

Figure 4 shows the effect of *sleep timer* on the energy consumption for varying activity, where the values of *λ_t_*, *λ_r_*, and *λ_f_* are changed with $\frac{1}{\mu_{t}}=\frac{1}{\mu_{r}}=\frac{1}{\mu_{f}}=\frac{1}{3600}h$. We note that simulation results only for steady state probabilities are presented but those for energy consumption are not presented in this paper since energy consumption is just a weighted sum of steady state probabilities, and thus, the validity of the analytical model for energy consumption is proved by just showing that for steady state probabilities. As can be expected from the result in Figure 3, the energy consumption decreases as the value of *sleep timer* increases, since the effect of *sleep* state becomes dominant, which has low power consumption. For a fixed value of *sleep timer*, energy consumption increases as the activity increases due to more transitions to *active* state with higher power consumption. We note that although the energy consumption decreases as the value of *sleep timer* increases, this also increases wakeup delay from *sleep* state to *listen* state. Therefore, an appropriate selection of *sleep timer* is needed to tradeoff between power consumption and wakeup delay.

**Figure 5.**
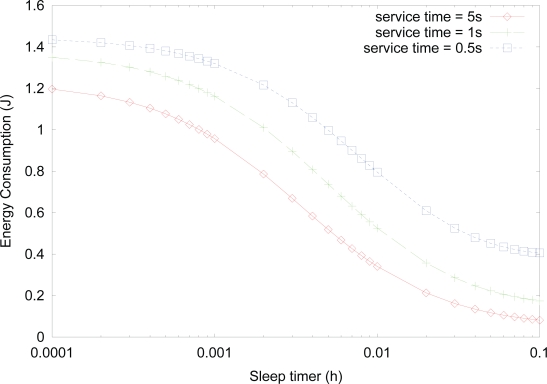
Effect of *sleep timer* on the energy consumption for varying service time.

Figure 5 shows the effect of *sleep timer* on the energy consumption for varying service time, where the value of activity is fixed as 0.1. Although the value of activity is the same, smaller service time, *i.e.*, higher data arrival, results in more state changes into *active* state. Therefore, the energy consumption increases as the service time for transmitting, receiving, or forwarding data decreases. This is because although the total service time for either transmitting, receiving, or forwarding data is the same for the same activity, the sensor node with higher data arrival rate has higher probability of *active* state, since the sensor node stays in *active* state additionally until the expiration of *active timer*, where the number of the expirations of *active timer* is higher for higher rate of data arrival.

### 4.3. Effect of Listen Timer, T_l_

**Figure 6.**
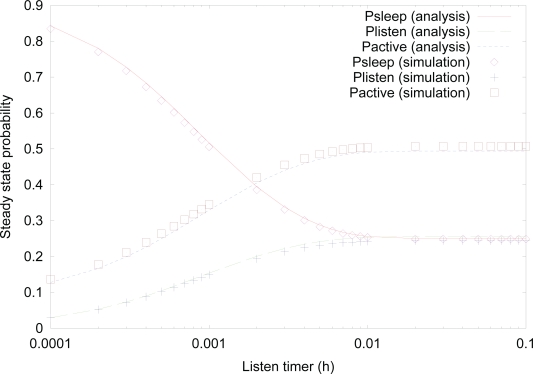
Effect of *listen timer* on the steady state probability.

Figure 6 shows the effect of *listen timer* on the steady state probability using both analysis and simulation. Similar to Figure 3, analysis results are well matched with simulation results. The probability of *sleep* state decreases, as the value of *listen timer* increases, since it is more likely that a sensor node stays in *listen* state more for higher value of *listen timer*, which is evident from the definition of *listen* timer. The probability of *active* state increases since it has more chances to make transition from *listen* state to *active* state due to data arrival, as the value of *listen timer* increases.

**Figure 7.**
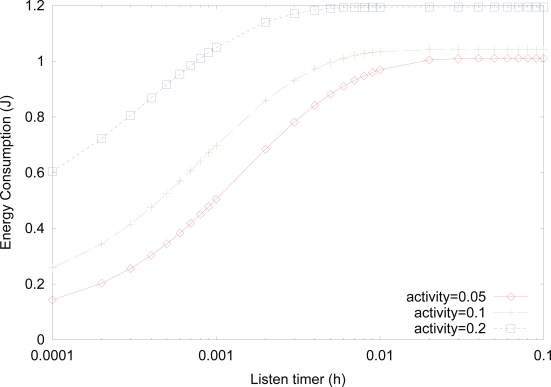
Effect of *listen timer* on the energy consumption for varying activity.

Figure 7 shows the effect of *listen timer* on the energy consumption for varying activity, where the values of *λ_t_*, *λ_r_*, and *λ_f_* are changed with $\frac{1}{\mu_{t}}=\frac{1}{\mu_{r}}=\frac{1}{\mu_{f}}=\frac{1}{3600}h$. As can be expected from the result in Figure 6, the energy consumption increases as the value of *listen timer* increases, since the probabilities of *listen* and *active* states, which have higher power consumption, increase. For a fixed value of *listen timer*, energy consumption increases as the activity increases due to more transitions into *active* state with higher power consumption. We note that although smaller value of *listen timer* is better, from the aspect of energy consumption, the smaller value of *listen timer* results in more transitions into *sleep* state. Since more transitions into *sleep* state result in higher wakeup delay, an appropriate selection of *listen timer* is needed to tradeoff between power consumption and wakeup delay.

**Figure 8.**
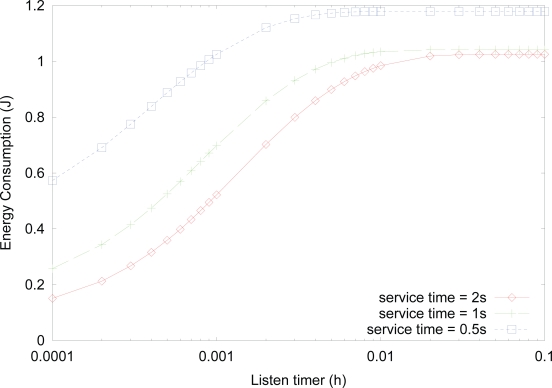
Effect of *listen timer* on the energy consumption for varying service time.

Figure 8 shows the effect of *listen timer* on the energy consumption for varying service time, where the value of activity is fixed to 0.1. Although the value of activity is the same, the energy consumption increases as the service time for transmitting, receiving, or forwarding data decreases due to the similar rationale, as in Figure 5.

### 4.4. Effect of Active Timer, T_a_

**Figure 9.**
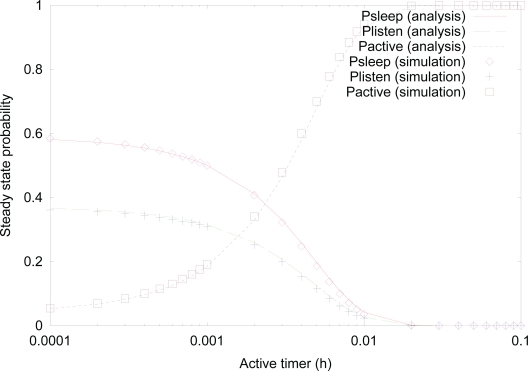
Effect of *active timer* on the steady state probability.

Figure 9 shows the effect of *active timer* on the steady state probability using both analysis and simulation. Similar to previous figures on steady state probabilities, analysis results are well matched with simulation results, as well. As can be expected, the probability of *active* state increases, as the value of *active timer* increases. On the other hand, the probability of *sleep* and *listen* states decreases, as the value of *active timer* increases. This is due to the fact that higher value of *active timer* results in less transitions to *sleep* state, and thus, the transition from *sleep* state to *listen* state is also less likely to occur.

**Figure 10.**
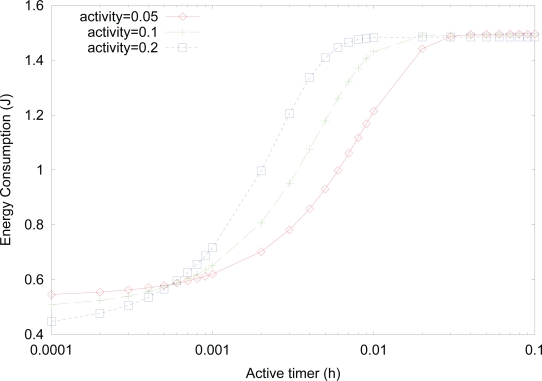
Effect of *active timer* on the energy consumption for varying activity.

Figure 10 shows the effect of *active timer* on the energy consumption for varying activity, where the values of *λ_t_*, *λ_r_*, and *λ_f_* are changed with $\frac{1}{\mu_{t}}=\frac{1}{\mu_{r}}=\frac{1}{\mu_{f}}=\frac{1}{3,600}h$. Since the probability of *active* state increases as the value of *active timer* increases, the energy consumption also increases since *active* state has more power consumption. For a fixed value of *active timer*, energy consumption generally increases as the activity increases, due to more transitions into *active* state with higher power consumption. On the other hand, for very small values of *active timer*, energy consumption decreases, even though the activity increases, because more transitions into *active* state due to higher activity also results in more transitions into *listen* state with very low power consumption from *active* state, after the fast expiration of small *active timer*.

**Figure 11.**
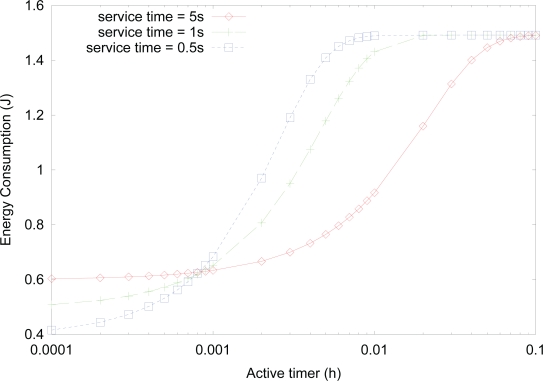
Effect of *active timer* on the energy consumption for varying service time.

Figure 11 shows the effect of *active timer* on the energy consumption for varying service time, where the value of activity is fixed as 0.1. Although the value of activity is the same, the energy consumption generally increases as the service time for transmitting, receiving, or forwarding data decreases due to the similar rationale, as in Figure 8. On the other hand, for very small values of *active timer*, energy consumption decreases as the service time increases, due to the similar rationale, as in Figure 10.

### 4.5. Effect of λ_t_

**Figure 12.**
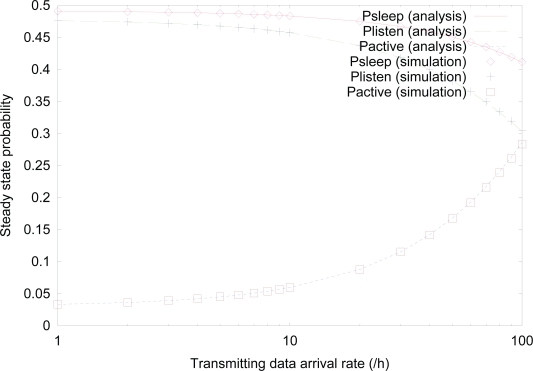
Effect of *λ_t_* on the steady state probability.

Figure 12 shows the effect of *λ_t_* on the steady state probability for *λ_f_* = *λ_r_* = 10/h using both analysis and simulation. Similar to previous figures on steady state probabilities, analysis results are well matched with simulation results, too. The steady state probability of *active* state sharply increases, as the value of *λ_t_* increases, which also increases the values of *λ_r_* and *λ_f_*. On the other hand, the steady state probabilities of both *sleep* and *listen* states decrease, as the value of *λ_t_* increases, due to more transitions to *active* state from these states.

**Figure 13.**
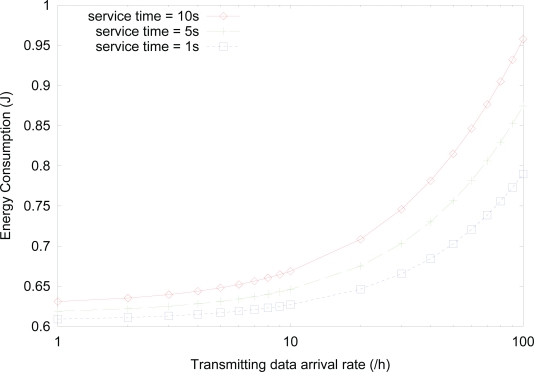
Effect of *λ_t_* on the energy consumption.

Figure 13 shows the effect of *λ_t_* on the energy consumption for varying service time, where the value of activity increases as the service time increases. As can be expected from Figure 12, the energy consumption also sharply increases, as the value of *λ_t_* increases, with the dominant effect of *active* state having higher power consumption. For a fixed value of *λ_t_*, power consumption also increases as the service time increases due to higher activity.

## 5. Conclusions and Further Works

In this paper, we developed an analytical methodology of state transition model of an energy conservation scheme based on duty cycling and derive both steady state probability of sensor node states and energy consumption analytically based on traffic characteristic and timer values. Then, the effects of *sleep timer*, *listen timer*, *active timer*, and traffic characteristics on the steady state probability and energy consumption have been analyzed.

From the numerical examples, it can be concluded that the steady state probability and thus, resulting energy consumption are very sensitive to the change of timer values and traffic characteristic, and thus, an appropriate selection of timer values taking into account of traffic characteristics is very important for efficient power conservation. The result of this paper can provide sensor network operators guideline for selecting appropriate timer values for efficient energy conservation, depending on traffic characteristic. We note that since the analytical methodology developed in this paper is based on a general energy conservation scheme based on duty cycling, it can be extended to other energy conservation schemes based on duty cycling with different sensor node states, without difficulty.

As further works, the effect of timer values and traffic characteristics on the other performance measures, such as the delay of data delivery and the number of retransmissions for successful routing will be analyzed for more thorough analysis of an energy conservation scheme based on duty cycling, using the analytical model developed in this paper. The extension of the proposed analytical model for system optimization or design of a new duty cycling scheme, such as traffic-aware duty cycling scheme based on dynamic adjustments of optimal timer values for varying traffic environments, will be studied in further works too.

## Figures and Tables

**Table 1.** Parameter values for timers and traffic characteristic.

Parameter	Value
*T_s_*	$\frac{10}{3600}h$
*T_l_*	$\frac{10}{3600}h$
*T_a_*	$\frac{10}{3600}h$
*ρ*	0.1
*w_f_*	10
$\frac{1}{\mu_{t}}$	$\frac{10}{3600}h$
$\frac{1}{\mu_{r}}$	$\frac{10}{3600}h$
$\frac{1}{\mu_{f}}$	$\frac{10}{3600}h$
*λ_t_*	$\frac{3600}{210}/h$
*λ_r_*	$\frac{3600}{21}/h$
*λ_f_*	$\frac{3600}{21}/h$

**Table 2.** Parameter values for power consumption.

Parameter	Value
ψ_1_	0.025 W
ψ_2_	1.155 W
ψ_3_	1.6 W
ψ_4_	1.2 W
ψ_5_	1.6 W
ψ_6_	1.5 W

## References

[1] Akyildiz I., Su W., Sankarasubramaniam Y., Cayirci E. (2002). A survey on sensor networks. IEEE Commun. Mag.

[2] Yick J., Mukherjee B., Ghosal D. (2008). Wireless sensor network survey. Elsevier Comput. Netw.

[3] Pantazis N.A., Vergados D.D. (2007). A Survey on Power Control Issues in Wireless Sensor Networks. IEEE Commun. Surv. Tutorials.

[4] Anastasi G., Conti M., Francesco M.D., Passarella A. (2009). Energy conservation in wireless sensor networks: A survey. Elsevier*Ad Hoc* Networks.

[5] Savvides A., Han C.C., Srivastava M. Dynamic fine-grained localization in *ad hoc* networks of sensors.

[6] Stemm M., Katz R.H. (1997). Measuring and reducing energy consumption of network interfaces in hand-held devices. IEICE Trans. Commun.

[7] Jones C.E., Sivalingam K.M., Agrawal P., Chen J.C. (2001). A survey of energy efficient network protocols for wireless networks. Wirel. Netw.

[8] Vuran M.C., Akan O.B., Akyildiz I.F. (2004). Spatio-temporal correlation: theory and applications for wireless sensor networks. Elsevier Comput. Netw.

[9] Chakrbarti A., Sabharwal B., Zazhang B. Using predictable observer mobility for power efficient design of sensor networks.

[10] Santi P. (2005). Topology control in wireless *ad hoc* and sensor network. ACM Comput. Surv.

[11] Xu Y., Heidemann J., Estrin D. Geography-informed energy conservation for *ad hoc* routing.

[12] Chen B., Jamieson K., Balakrishnan H., Morris R. (2002). Span: An energy-efficient coordination algorithm for topology maintenance in *ad hoc* wireless networks. Wirel. Netw.

[13] Xu Y., Heidemann J., Estrin D. (2000). Adaptive Energy-conservation Routing for Multi-hop *Ad Hoc* Networks.

[14] Schugers C., Tsiatsis V., Srivastava M.B. STEM: topology management for energy efficient sensor networks.

[15] Keshavarzian A., Lee H., Venkatraman L. Wakeup scheduling in wireless sensor networks.

[16] Gao Q., Blow K.J., Holding D.J., Marshall I. (2005). Analysis of energy conservation in sensor networks. Wirel. Netw.

[17] Rai V., Mahapatra R.N. Lifetime modeling of a sensor network.

[18] Noori M., Ardakani M. A probability model for lifetime of event-driven wireless sensor networks.

[19] Ross S.M. (1996). Stochastic Processes.

